# Decoupling of Internal and External Workload During a Marathon: An Analysis of Durability in 82,303 Recreational Runners

**DOI:** 10.1007/s40279-022-01680-5

**Published:** 2022-05-05

**Authors:** Barry Smyth, Ed Maunder, Samuel Meyler, Ben Hunter, Daniel Muniz-Pumares

**Affiliations:** 1 Insight Centre for Data Analytics, School of Computer Science, University College Dublin, Dublin, Ireland; 2 Sports Performance Research Institute New Zealand, Auckland University Technology, Auckland, New Zealand; 3 School of Life and Medical Sciences, University of Hertfordshire, Hatfield, AL10 9AB UK

## Abstract

**Aim:**

This study characterised the decoupling of internal-to-external workload in marathon running and investigated whether decoupling magnitude and onset could improve predictions of marathon performance.

**Methods:**

The decoupling of internal-to-external workload was calculated in 82,303 marathon runners (13,125 female). Internal workload was determined as a percentage of maximum heart rate, and external workload as speed relative to estimated critical speed (CS). Decoupling magnitude (i.e., decoupling in the 35–40 km segment relative to the 5–10 km segment) was classified as low (< 1.1), moderate (≥ 1.1 but < 1.2) or high (≥ 1.2). Decoupling onset was calculated when decoupling exceeded 1.025.

**Results:**

The overall internal-to-external workload decoupling experienced was 1.16 ± 0.22, first detected 25.2 ± 9.9 km into marathon running. The low decoupling group (34.5% of runners) completed the marathon at a faster relative speed (88 ± 6% CS), had better marathon performance (217.3 ± 33.1 min), and first experienced decoupling later in the marathon (33.4 ± 9.0 km) compared to those in the moderate (32.7% of runners, 86 ± 6% CS, 224.9 ± 31.7 min, and 22.6 ± 7.7 km), and high decoupling groups (32.8% runners, 82 ± 7% CS, 238.5 ± 30.7 min, and 19.1 ± 6.8 km; all *p* < 0.01). Compared to females, males’ decoupling magnitude was greater (1.17 ± 0.22 vs. 1.12 ± 0.16; *p* < 0.01) and occurred earlier (25.0 ± 9.8 vs. 26.3 ± 10.6 km; *p* < 0.01). Marathon performance was associated with the magnitude and onset of decoupling, and when included in marathon performance models utilising CS and the curvature constant, prediction error was reduced from 6.45 to 5.16%.

**Conclusion:**

Durability characteristics, assessed as internal-to-external workload ratio, show considerable inter-individual variability, and both its magnitude and onset are associated with marathon performance.

## Key Points

**Table:** 

The decoupling of internal-to-external workload ratio can be used to quantify the ‘durability’ of endurance athletes during long-duration exercise. We used the decoupling of internal (i.e., heart rate) and external (i.e., grade-adjusted speed) workloads, expressed as a ratio indexed to the 5–10 km segment, to quantify the ‘durability’ of > 80,000 marathon runners. Specifically, we assessed the relationship between the magnitude and onset of this decoupling with marathon performance.
There was a large inter-individual variation in the magnitude and onset of decoupling. However, when classified as low, moderate and high decoupling, athletes experiencing low decoupling had better marathon performance. Moreover, models of marathon performance were improved when both magnitude and onset decoupling are included.
The data presented herein suggest that the decoupling of internal-to-external workload ratio should be taken into consideration during long-duration exercise, as it can contribute to explain marathon performance.

## Introduction

Marathon running has been the subject of considerable interest in recent years, and it is generally accepted that multiple factors can affect its performance [1–4]. For example, models explaining marathon performance have typically considered three physiological traits: the maximum oxygen uptake ($\dot{V}{\text{O}}_{2\max }$), oxygen cost of movement (i.e., running economy), and the fraction of $\dot{V}{\text{O}}_{2\max }$ that can be maintained for the duration of the marathon [2, 5, 6]. Combined, these physiological traits result in a ‘performance metabolic rate’, the highest oxidative metabolic rate that can be sustained for the marathon. Critical speed (CS) is the physiological threshold delineating the heavy- and severe-intensity domains, and therefore defines the point at which the maximal metabolic steady-state (MMSS) can be attained, and exercise can be supported mainly from oxidative metabolism [7–10]. It is worth noting that several other terms or approaches have been suggested to correspond with, or permit the approximation of, the MMSS including ventilatory or respiratory thresholds, or thresholds derived from blood lactate concentration, such as the maximal lactate steady state [8]. Indeed, criticism of the CS model has been levelled as the concordance of estimates with the MMSS can be dependent on the methodology used [11, 12]. Since CS (and its analogous critical power) demarcates the boundary between heavy and severe exercise domains [8, 13, 14], and thus represents a marker of the MMSS, it follows that CS shows a strong association with endurance performance—including marathon performance [15, 16].

An interesting finding from studies investigating the ability of CS to predict marathon performance [15, 16] was that faster athletes appear to complete the marathon at higher speeds relative to their CS than slower athletes. Thus, elite marathon runners with an average finishing time of ~ 2 h and 5 min could complete the marathon at ~ 96% of their CS. However, well-trained athletes with an average time of ~ 2 h and 30 min completed the marathon at ~ 93% CS, whereas recreational athletes with an average marathon time of ~ 4 h managed to complete the marathon at ~ 79% CS. A plausible explanation of this apparently linear decrease in marathon speed, relative to CS, with increasing marathon times is that physiological attributes crucial in marathon performance, reflected as the CS, represent the maximum ability of a fully rested athlete, but such physiological attributes deteriorate during prolonged exercise, such as a marathon. Clark et al. [17, 18] recently reported that critical power, the cycling equivalent of CS, decreased by ~ 10–15% following 2 h of heavy exercise. Therefore, if a similar decrease in CS also occurs with prolonged running, it is plausible that marathon runners who start the marathon at speeds close to but fractionally below their CS transition into severe intensity exercise (above CS) during a marathon, even if the speed is maintained constant throughout the race. It is plausible that better athletes may be able to preserve physiological traits, and thus maintain speeds closer to CS. Indeed, it has recently been suggested that durability, defined as deterioration in physiological characteristics over time during prolonged exercise [19], should be taken into consideration during physiological and performance profiling.

The aims of this study, therefore, were to (i) characterise the decoupling of internal-to-external workload during a marathon in a large cohort of recreational runners; and (ii) investigate whether the magnitude and time of onset of the decoupling could predict marathon performance, and whether taking into consideration the decoupling improved predictions derived from CS alone. Furthermore, given recent reports highlighting the differences in fatigability between males and females [20], which may contribute to the observed sex differences in endurance performance [20, 21], we report and compare decoupling traits for male and female athletes separately. We hypothesised that marathon runners with faster finishing times would exhibit reduced decoupling of internal-to-external workload ratio compared to runners with slower finishing times. Specifically, we hypothesise that athletes exhibiting low decoupling and/or late onset in decoupling of internal-to-external workload ratio will be able to perform closer to their CS. Therefore, we hypothesised that by combining CS with estimations of the magnitude of the decoupling of internal-to-external workload ratio, models of marathon performance would be improved. Finally, we hypothesised that the magnitude of decoupling would be lower in female athletes compared to that observed in their male counterparts.

## Methods

### Dataset

A large dataset of recreational runners was made available to the authors by the running platform Strava^®^ (Strava, Inc., San Francisco, CA, USA) under limited research license. The dataset contained anonymised data and, therefore, the ethics boards of all institutions (Auckland University of Technology, University College Dublin, and University of Hertfordshire) deemed the study exempt from ethical approval. Athletes uploaded the data from training sessions, collected through smartphones or other devices (e.g., running pods), into the running platform. The dataset consisted of time, location, distance, and elevation data sampled at 100 m intervals. In addition, heart rate (HR) was available from all training sessions. HR data was processed in a similar way to running data, and thus averaged at 100 m intervals. The characteristics of the dataset used in the current study are provided in Table 1. There were 82,303 runners (~ 16% female) included in this study, for whom training data were available for the ~ 4 months preceding a marathon. For all athletes, the dataset contained at least one marathon race. In an attempt to identify genuine marathons, we identified sessions that matched a marathon distance (i.e., 42.2 km), but also contained multiple runners starting at the same time and location. This approach provided a series of candidate marathon races that were manually identified, so that genuine marathon races were differentiated from ‘practice’ marathons.

**Table 1:** Descriptive statistics of the dataset

	F	M	All
Athletes (*n*)	13,125	69,178	82,303
Age (y)	37 ± 8	40 ± 26	39 ± 24
Finish time (min)	245.2 ± 29.6	223.3 ± 32.5	226.8 ± 33.1
Training sessions (*n*)	72 ± 33	70 ± 34	70 ± 34
Weeks (*n*)	18.2 ± 2.6	18.2 ± 2.5	18.2 ± 2.5
Training frequency (sessions·wk^−1^)	3.9 ± 1.6	3.8 ± 1.7	3.8 ± 1.7
Training volume (km·wk^−1^)	40.9 ± 15.74	43.0 ± 17.9	42.7 ± 17.6

*F* female runners, *M* male runners, *All* all runners

### Critical Speed and *D*′ Determination

Critical speed and *D*′, the curvature constant of the speed-duration relationship that represents running capacity above CS, were estimated from raw training data, as previously described [15]. In brief, raw data from all training sessions for each athlete were first converted to grade-adjusted speed. This approach accounts for changes in elevation, for instance when running uphill or downhill, and is described in more detail elsewhere [15, 22]. The fastest grade-adjusted speed observed in any training session for each athlete was recorded for a range of distances (400, 800, 1500, 3000, and 5000 m), and then used to construct the distance-time relationship according to a linear model of distance and time [23]. For each athlete, the slope of this line was considered CS, and the intercept of the line the curvature constant, *D*′ [23].

### Durability and Decoupling

Each marathon was divided into eight 5-km segments plus the final 2 km of the race, and the decoupling of internal-to-external workload ratio was calculated for each segment. The internal workload was determined as a percentage of maximum HR (HR_max_). The HR_max_ for the cohort was given as 178 ± 18 beats per min (bpm) and 187 ± 8 bpm using an age-predicted calculation [24] and the highest HR recorded in any training session, respectively. Therefore, HR_max_ was defined as the highest HR recorded in any training session for each runner. The external workload was determined as the speed, relative to CS, during the recorded marathon. The first (0–5 km) and last (40–42.2 km) segments of the race were excluded to avoid possible artefacts caused by sudden changes in pace in the first and last few kms of the race, respectively. The decoupling observed in the last 5 km segment of the race (35–40 km) was used to determine the overall magnitude of the decoupling experienced by each athlete, and expressed relative to the 5–10 km segment. Thus, a decoupling of 1.15 indicates that internal-to-external ratio (ratio between %HR_max_ and %CS) was 15% greater in the 35–40 km segment compared to that observed in the 5–10 km segment of the race. To estimate the onset of decoupling, the race segment from which decoupling remained consistently (i.e., for the remaining of the race) above 1.025 was calculated for each athlete, focusing on the race segments from 10 to 40 km. We converted this race segment into an estimated distance by calculating the mid-point of the segment. Thus, if a decoupling > 1.025 was first detected in the 20–25 km segment of the marathon and sustained to the 35–40 km segment, then the onset was assumed to be at 22.5 km. The distance at which decoupling was first observed was converted to time of onset using average running speed. If a decoupling > 1.025 was not detected at all for a runner, the onset was assumed to be their either 42.2 km or their finish-time, as appropriate, to represent a runner completing the marathon without decoupling.

### Data Analysis

Athletes experiencing a decoupling < 1.1 in the last segment of the race were classified as low decoupling, a decoupling ≥ 1.1 but < 1.2 was considered as moderate, and if the decoupling was ≥ 1.2 it was deemed as high decoupling [19]. In order to investigate whether decoupling experienced by an athlete contributed to explain marathon performance, the correlation between key decoupling characteristics (i.e., magnitude and the onset of decoupling) and absolute (marathon time) and relative (marathon speed relative to CS) marathon performance was determined. To calculate these correlations, athletes were grouped based on their relative performance (in 5% bins, from 70% CS to 90% CS) and absolute performance (in 30 min bins, from 150 to 270 min). Finally, a SciKit learn [Python (Python Software Foundation, Wilmington, DA, USA)] implementation of a gradient boosting regressor [25] was used to predict marathon performance based on CS and D′; this regressor was configured to use n = 5,000 estimators and a learning rate of 0.005 [25]. This approach has already been shown to predict performance with relative success (~ 7% error, Ref. [15]). Therefore, the model was modified to consider CS and *D*′ as well as durability traits, namely the magnitude and onset of the decoupling. Mean values between sexes and decoupling groups (low vs. moderate, moderate vs. high) were compared with a Welch's *t*-test (which does not assume equal population variance), and significance was accepted at *p* < 0.01. Cohen’s *d* was used as a measure of effect-size, and interpreted as very small (0.01), small (0.20), medium (0.50), large (0.80), very large (1.20) and huge (2.00) [26]. Results are reported as mean ± standard deviation.

## Results

### Marathon Performance and Critical Speed

The overall marathon performance and decoupling characteristics of the athletes within the dataset are presented in Table 2. Overall, the marathon was completed at 3.17 ± 0.47 m·s^−1^, and thus marathon time was ~ 3 h and 47 min ± 33 min. The CS and *D*′, estimated from raw training data corresponded to 3.72 ± 0.48 m·s^−1^ and 196 ± 90 m, respectively, and therefore the average marathon speed corresponded to 85 ± 7% of CS. Male runners had ~ 10% superior marathon performance and CS compared to female runners (both *p* < 0.01), but females were able to complete the marathon at speeds closer to their CS (87 ± 6 vs. 85 ± 7% CS, respectively; *p* < 0.01, *d* = 0.23).

**Table 2:** Marathon performance and decoupling characteristics in 83,303 recreational runners

	ALL			F			M				M v F	
		Sig	*d*		Sig	*D*		Sig		*d*	Sig	*d*
Marathon time (min)												
Low decoupling	217.3 ± 33.1	^a^	0.23	240.5 ± 29.9	^a^	0.22	211.1 ± 31.1	^a^		0.31	*	0.95
Moderate decoupling	224.9 ± 31.7	^b^	0.43	246.9 ± 28.9	^b^	0.21	220.7 ± 30.4	^b^		0.53	*	0.87
High decoupling	238.5 ± 30.7	^c^	0.66	252.9 ± 28.0	^c^	0.42	236.9 ± 30.6	^c^		0.84	*	0.53
All athletes	226.8 ± 33.1			245.2 ± 29.6			223.3 ± 32.5				*	0.68
Marathon speed (m·s^−1^)												
Low decoupling	3.31 ± 0.50	^a^	0.26	2.97 ± 0.38	^a^	0.22	3.40 ± 0.49	^1^		0.33	*	0.92
Moderate decoupling	3.19 ± 0.45	^b^	0.44	2.89 ± 0.36	^b^	0.21	3.25 ± 0.45	^2^		0.53	*	0.83
High decoupling	3.00 ± 0.41	^c^	0.68	2.82 ± 0.34	^c^	0.42	3.02 ± 0.41	^3^		0.85	*	0.51
All athletes	3.17 ± 0.47			2.91 ± 0.37			3.22 ± 0.48				*	0.67
Critical speed (m·s^−1^)												
Low decoupling	3.78 ± 0.51	^a^	0.14	3.39 ± 0.40	^1^	0.09	3.89 ± 0.48	^1^		0.23	*	1.10
Moderate decoupling	3.71 ± 0.47	^b^	0.11	3.35 ± 0.39			3.78 ± 0.45	^2^		0.19	*	0.98
High decoupling	3.67 ± 0.44	^c^	0.25	3.36 ± 0.38	^3^	0.08	3.70 ± 0.43	^3^		0.42	*	0.80
All athletes	3.72 ± 0.48			3.37 ± 0.39			3.79 ± 0.46				*	0.93
Marathon speed (/CS)												
Low decoupling	0.88 ± 0.06	^a^	0.25	0.88 ± 0.06	^a^	0.24	0.88 ± 0.07	^a^		0.25	*	0.04
Moderate decoupling	0.86 ± 0.06	^b^	0.59	0.86 ± 0.06	^b^	0.36	0.86 ± 0.06	^b^		0.61	*	0.07
High decoupling	0.82 ± 0.07	^c^	0.84	0.84 ± 0.06	^c^	0.60	0.82 ± 0.07	^c^		0.85	*	0.34
All athletes	0.85 ± 0.07			0.87 ± 0.06			0.85 ± 0.07				*	0.23
Decoupling magnitude (AU)												
Low decoupling	1.01 ± 0.18	^a^	1.00	1.02 ± 0.12	^1^	1.33	1.01 ± 0.2	^1^		0.95		
Moderate decoupling	1.15 ± 0.03	^b^	1.07	1.14 ± 0.03	^2^	1.67	1.15 ± 0.03	^2^		1.03	*	0.11
High decoupling	1.33 ± 0.24	^c^	1.49	1.31 ± 0.16	^3^	2.18	1.33 ± 0.24	^3^		1.42	*	0.07
All athletes	1.16 ± 0.22			1.12 ± 0.16			1.17 ± 0.22				*	0.22
Decoupling onset (km)												
Low decoupling	33.4 ± 9.0	^a^	1.32	32.9 ± 9.8	^1^	1.26	33.6 ± 8.7	^a^		1.35	*	0.25
Moderate decoupling	22.6 ± 7.3	^b^	0.49	21.7 ± 7.6	^2^	0.34	22.8 ± 7.2	^b^		0.52	*	0.15
High decoupling	19.1 ± 6.8	^c^	1.79	19.1 ± 7.3	^3^	1.01	19.2 ± 6.7	^c^		1.86		
All athletes	25.2 ± 9.9			26.3 ± 10.6			25.0 ± 9.8				*	0.13
Decoupling onset (min)												
Low decoupling	170.1 ± 53.8	^a^	1.15	185.1 ± 61.1	^1^	1.16	166.1 ± 50.9	^a^		1.14	*	0.36
Moderate decoupling	115.2 ± 40.7	^b^	0.43	121.0 ± 45.8	^2^	0.35	114.1 ± 39.6	^b^		0.43	*	0.17
High decoupling	98.4 ± 37.2	^c^	1.54	105.3 ± 42.4	^3^	1.43	97.6 ± 36.5	^c^		1.56	*	0.21
All athletes	128.6 ± 54.3			147.3 ± 63.6			125.1 ± 51.6				*	0.41

ALL represents all athletes in the dataset, whereas F and M represent data from female and male athletes, respectively. The column ‘F v M’ shows whether there was a difference between male and females, where the symbol * depicted a significant difference (*p* < 0.01) and the corresponding effect size

The subscripts ^a^, ^b^ and ^c^ indicate whether a significant difference (*p* < 0.01) was observed between low vs. moderate decoupling, moderate vs. high decoupling, and low vs. high decoupling, respectively. Decoupling magnitude represents the internal-to-external workload ratio in the 35–40 km segment, and is reported in arbitrary units (AUs)

### Internal-to-External Workload Decoupling During Marathon Running

**Fig. 1:**
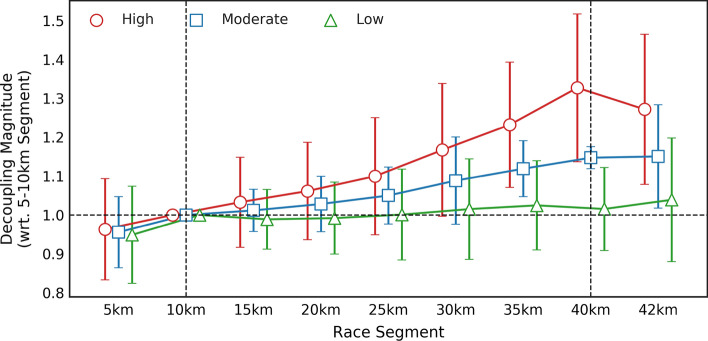
Time-course of the decoupling of internal-to-external workload for athletes with low, moderate, and high decoupling. Low, moderate and high decoupling was defined as athletes with a decoupling < 1.1, between 1.1 and 1.2, and > 1.2 in the 35–40 km segments. Decoupling is expressed relative to the 5–10 km segment of the marathon

The average decoupling experienced in the 35–40 km segment was 1.16 ± 0.22. However, there was considerable inter-individual variation. Out of 82,303 runners, 34.5% (28,404 runners) exhibited low decoupling (decoupling < 1.1 in the 35–40 km segment), 32.7% (26,879 runners) moderate decoupling (≥ 1.1 but < 1.2), and 32.8% (27,020 runners) were classified as high decoupling (≥ 1.2). The time-course of decoupling for the low, moderate, and high decoupling groups over the course of a marathon is shown in Fig. 1.

The overall magnitude of decoupling was greater for males compared to female runners (1.17 ± 0.22 vs. 1.12 ± 0.16; *p* < 0.01, *d* = 0.22). Male runners were relatively evenly distributed in the low, moderate and high decoupling groups (32.3%, 32.6% and 35.1%, respectively), whereas their female counterparts were more frequently classified as low and moderate decoupling compared to high decoupling (46.1%, 33.2% and 20.7%, respectively).

**Fig. 2:**
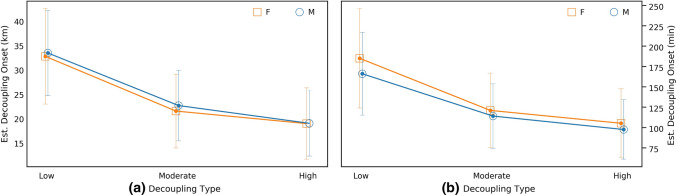
Estimated onset of decoupling during a marathon and decoupling type (low, moderate and high), for male (M) and female (F) runners. The filled circles in the high decoupling indicate a male—female difference (*p* < 0.01)

The onset of decoupling, when runners first exhibited a continuous decoupling > 1.025 sustained to the end of the marathon, occurred after 25.2 ± 9.9 km. However, there were differences for each decoupling group (Table 2), whereby the onset of the decoupling occurred later in the low decoupling group, compared to the moderate and high decoupling groups. The onset of decoupling occurred first in male runners, irrespective of the magnitude of decoupling experienced (low, moderate or high), as shown in Table 2. When the onset of decoupling was expressed as time, males also experienced earlier decoupling compared to female runners (147.3 ± 63.6 vs. 125.1 ± 51.6 min, respectively; *p* < 0.01, *d* = 0.41). This held true for all decoupling groups (low, moderate and high decoupling; Table 2, Fig. 2).

### Internal-to-External Workload Decoupling and Marathon Performance

**Fig. 3:**
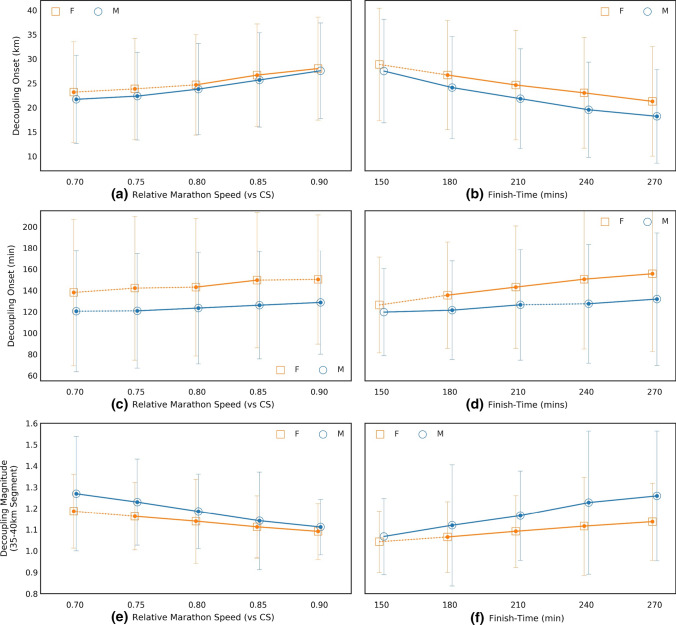
The onset (distance and time) and the magnitude of the decoupling of internal-to-external workload ratio relative to marathon performance, where marathon performance is calculated: **a** relative CS, and **b** in absolute units (min). Estimated onset of the decoupling of internal-to-external workload relative to marathon performance, where marathon performance is calculated: **c** relative CS, and **d** in absolute units (min). Filled markers indicate a significant difference between male and female runners (*p* < 0.01) and a solid line between two makers indicates a statistically significant difference between consecutive pace bins (*p* < 0.01)

Both relative marathon performance (marathon speed relative to CS) and absolute marathon performance (marathon finish time) exhibited a strong association with the magnitude of the decoupling. Athletes exhibiting lower decoupling magnitude completed the marathon at a higher percentage of CS (*p* < 0.01, *R*^2^ = − 0.97) and faster marathon time (*p* < 0.01, *R*^2^ = 0.99, Fig. 3). Similarly, a strong association was observed between the onset of decoupling and marathon performance (Fig. 3), whereby athletes who experienced decoupling early during the marathon were able to complete the marathon at a higher fraction of their CS (*p* < 0.01, *R*^2^ = 0.92), and had faster marathon times (*p* < 0.01, *R*^2^ = − 0.99, Fig. 3a, b).

### Prediction of Marathon Performance

**Fig. 4:**
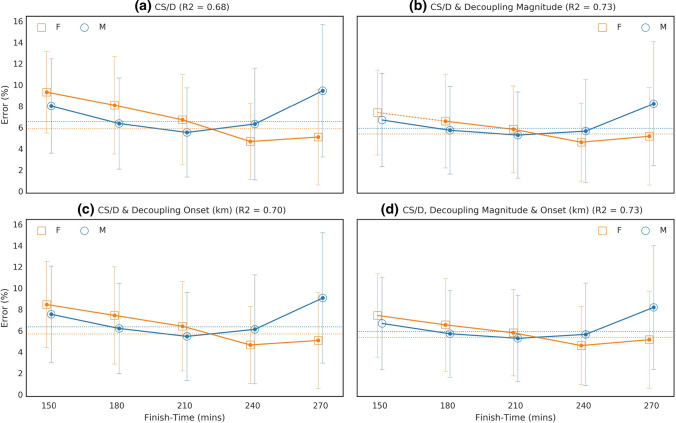
Error associated with predictions of marathon performance derived from **a** CS and *D’* only, **b** CS and *D’* plus the magnitude of the decoupling, and **c** CS and *D’* plus the decoupling degree and time to decoupling onset. The error is calculated as the mean absolute difference between the predicted finish-time and the actual finish-time as a fraction of actual finish-time for each finish-time group and the dotted lines show the mean error for male and females for all finish-times. In (**a**) a filled marker indicates a difference between the corresponding male and female means (*p* < 0.01), and a solid line between two makers indicates a difference between relative pace segments (*p* < 0.01). The overall *R*^2^ value for each finish-time is also shown

**Fig. 5:**
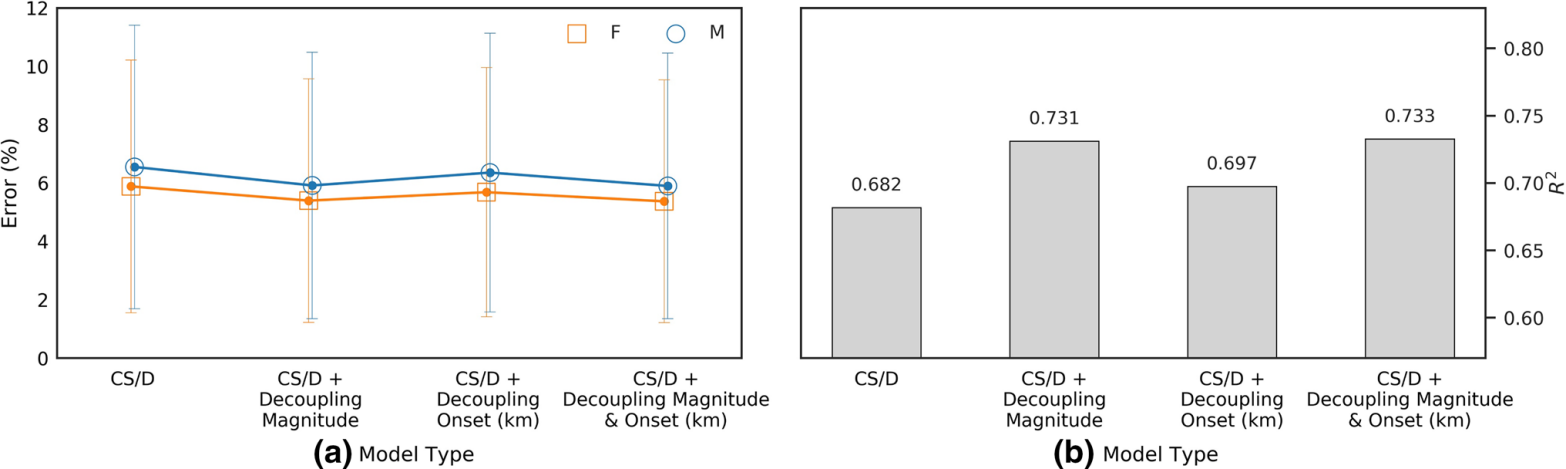
Overall performance of different models based exclusively on CS and *D*′, as well as parameters related to the decoupling of the internal-to-external workload ratio

Marathon performance was predicted with 6.45% error using a model that included CS and *D*′. Incidentally, marathon predictions based exclusively on CS presented with 6.62% error. However, including either the magnitude of the decoupling in the 35–40 km segment or the decoupling onset time reduced this error to 5.85% and 5.90%, respectively, which corresponds to relative improvements of 9.3% or 8.5%, respectively (see Fig. 4). When both magnitude and time of onset are included (alongside CS and *D*′), prediction error falls to 5.16%, which represents an overall improvement of 20.00% compared to the model using CS and *D*′ only. Overall, the prediction error was lower for female athletes (*p* < 0.01), irrespective of the model used (Fig. 5).

## Discussion

The primary aim of the present study was to explore the durability characteristics of a large, heterogenous group of recreational runners by calculating the decoupling of internal-to-external workload ratio during marathon running. In addition, we investigated whether the overall magnitude and onset of decoupling experienced by runners contributed to marathon performance, and whether these results were different in male and female runners. The main findings were that athletes experienced a ~ 1.16 (~ 16%) decoupling between HR and speed in marathon running, which started after 25.2 ± 9.9 km. However, there was large inter-individual variability, and runners could be classified into low, moderate and high decoupling groups. We found that runners in the low decoupling group completed the race at a higher percentage of their CS, with a faster overall time, and had a later onset of decoupling. Moreover, whilst CS and *D*′ were able to predict marathon performance, a model that incorporates durability characteristics (i.e., magnitude and onset of the decoupling) reduced the prediction error by 20%. Female runners exhibited a better durability profile, as the decoupling exhibited lower magnitude and later onset than that observed in male runners. These findings suggest that durability characteristics, such as its magnitude and onset, should be taking into consideration in marathon running because both parameters were associated with marathon performance. Moreover, the results from this study indicate that female runners experience less decoupling than their male counterparts.

### Inter-Individual Variation in Decoupling Characteristics

The large sample of recreational marathon runners analysed in the current study experienced internal-to-external workload decoupling of ~ 1.16, which indicates that the ratio between internal workload (HR) and external workload (grade-adjusted speed, relative to CS) increased by ~ 16% throughout a marathon. However, there was considerable inter-individual variability in the magnitude of decoupling. Athletes were classified, based on the magnitude of the decoupling observed in the last 5 km segment of the marathon, as low, moderate and high decoupling, as previously suggested [19]. Despite this being an arbitrary classification, we found a remarkably even distribution, and each of the three decoupling groups contained ~ 33% of athletes in the sample. Such inter-individual variability in the magnitude of decoupling supports consideration of durability in physiological profiling and performance modelling, as resilience to exercise-induced shifts in intensity domain transitions may contribute to performance capabilities in the latter stages of prolonged events [17–19].

Prolonged exercise, such as marathon running, necessitates a physiological steady state, and thus is typically performed at intensities close to, but below, CS [15, 16]. Exercise at intensities that exceed CS (or its cycling analogous, critical power) results in an inexorable increase in the concentration of muscle metabolites, such as hydrogen ions and inorganic phosphate, until an intolerable threshold is reached coinciding with the depletion of *D*′ and the attainment of $\dot{V}{\text{O}}_{2\max }$, which, ultimately, results in task failure soon afterwards [9, 27, 28]. Alternatively, exercise may be continued after the depletion of *D*′, but the intensity of exercise must remain below CS [29]. Previous studies have demonstrated that the power profile [30], CS [17, 18] and endurance performance [31] decrease with prolonged, submaximal exercise. Combined, the results from these studies and the data presented herein suggest that it is inappropriate to rely exclusively on physiological traits determined in fully rested state athletes to predict endurance performance. It is unlikely that such characteristics, determined at rest, remain constant during prolonged exercise, or that they deteriorate at a constant rate. Instead, athletes appear to exhibit different abilities to preserve their physiological abilities during prolonged exercise. Thus, monitoring the durability of athletes (e.g., by monitoring the decoupling of internal-to-external workload) should be taken into consideration in physiological profiling, when prescribing prolonged exercise or aiming to predict endurance (e.g., marathon) performance.

### Decoupling Characteristics and Marathon Performance

Previous studies have shown that CS is a strong predictor of marathon performance, with elite marathon runners’ best performances completed at 96% CS [16], and faster recreational marathon runners also completing marathons at speeds close to (> 90%), but below, CS [15]. In the present study, athletes in the low decoupling group were able sustain a higher fraction of their CS throughout the marathon, which also occurred later in the marathon. The results from the present study demonstrate that marathon runners who exhibited superior durability (i.e., had low decoupling) were also able to run closer to their CS, and also able to complete the marathon faster.

The onset of decoupling was estimated to occur when a decoupling of at least 1.025 was first detected. This is, again, an arbitrary threshold representing a 2.5% increase in internal-to-external workload ratio. However, we found that this approach of detecting the onset of decoupling was also associated with marathon performance (Fig. 3). Athletes exhibiting low decoupling were able to complete a further ~ 14 km of the marathon without signs of physiological deterioration (Table 2). Moreover, when the onset of decoupling was expressed as time, overall results indicate that decoupling is first observed ~ 128 min into the race (see Table 2). Clark et al. [17] reported that a decrease in critical power was observed following 2 h of cycling at moderate intensities, but not after 80 min. In the present study, however, the onset of decoupling was detected ~ 80 min later in the low decoupling groups compared to the low decoupling group (~ 105 vs. 185 min, see Table 2). Overall, this study shows that both magnitude of decoupling and onset of decoupling, expressed as distance covered or time elapsed before it was first detected, were associated with marathon performance.

Critical speed denotes the highest sustainable oxidative metabolic rate, and thus is strongly associated with endurance performance. Indeed, previous studies have shown that marathon performance can be predicted with ~ 7% error using models derived from CS [15]. Similarly, in the present study marathon performance was predicted with 6.45% error using a model that included CS and *D*′. The addition of durability traits to this model, namely its magnitude and onset, reduced the prediction error to 5.16%, a 20% improvement in accuracy. Therefore, the data presented in the current study support that models aiming to predict marathon performance, and more generally models of endurance performance, should take into consideration the durability of physiological traits.

### Mechanisms Underpinning Decoupling

There are several factors that can explain the decoupling of internal-to-external workload decoupling. The mechanisms explaining the inter-individual variability in durability characteristics may be related to skeletal muscle fibre type characteristics given type I fibres are more resilient to exercise-induced loss of mechanical efficiency [32]. Therefore, the muscle metabolic cost of producing a given running speed may be better maintained during marathon running in athletes with a greater proportion of type I fibres, and therefore reduced decoupling between internal and external work as the race progresses. Similarly, the availability of proteins involved in management of cellular stress, such as the heat shock proteins [33], may promote durability characteristics by improving the capacity to manage the cellular stress generated during prolonged exercise [34]. Durability characteristics may also be related to mitochondrial protein content, as a larger mitochondrial pool may spread the oxidative burden of demanding exercise and therefore reduce mitochondrial damage at the level of the individual mitochondrion during prolonged exercise. These physiological mechanisms remain speculative and warrant attention from laboratory-based investigations of the determinants of durability characteristics.

Further to purely physiological mechanisms, it could be postulated that runners with greater durability are able to preserve a more economical pattern of running throughout the marathon. The greatest sustainable running speed is strongly mediated by running economy (e.g., references [1, 5]). However, the O_2_ cost of running has been shown to increase concomitantly over increased distances [35]. Elevated levels of markers of muscular fatigue and skeletal muscle damage can interfere with contractile mechanisms through inhibitory effects on α-motoneurons by activating fatigue-sensitive afferent fibres [36]. Consequently, during periods of prolonged running the force output during the push off phase has been shown to be reduced. Indeed, running induced fatigue has been shown to alter kinematics [37], kinetics [38], as well as stride dynamics [39, 40]. Resultant compensatory alterations in gait pattern to maintain running speed may result in an upward drift in $\dot{V}{\text{O}}_{2}$, and an increase in internal workload at a given running speed. However, compensatory movement patterns observed alongside and increase in $\dot{V}{\text{O}}_{2}$ have been shown to be highly variable between runners [41]. Furthermore, it is important to acknowledge the extent of muscular fatigue will be dependent on the intensity domain in which exercise is performed. Therefore, further investigations are warranted to elucidate whether diminished running economy is a cause or a consequence of durability characteristics.

Decoupling was quantified as the internal-to-external ratio [19], and therefore decoupling could represent an increase in internal workload (i.e., HR), decrease in external workload (i.e., speed), or both. In the current dataset, speed fell following the onset of decoupling by 11.3%, whilst the HR remained constant throughout the marathon, and only increased by 1.6% (or ~ 2 bpm) since decoupling was first detected. These data suggest that during a marathon, a ‘mirror image’ of the slow component was present, whereby workload has to be decreased in order to maintain a constant $\dot{V}{\text{O}}_{2}$ [42] or HR [43] during prolonged, submaximal exercise. Therefore, factors typically associated with the slow component (e.g., mainly metabolic requirements of fatiguing muscle fibres and additional recruitment of motor units with lower efficiency, see [44] for a review) may also have contributed to the observed decoupling of internal-to-external workload ratio.

### Female Runners Exhibit Less Decoupling

The results of the present study demonstrate that females displayed a lower magnitude and later onset of decoupling than males (Fig. 2, Table 1). Moreover, there were over twice as many female athletes classified as low decoupling than high decoupling. Previous studies have shown that physiological thresholds that demarcate the exercise intensity domains are typically positioned at a higher percentage of $\dot{V}{\text{O}}_{2\max }$ in females [45]. The data from the current study indicate that, in addition, female runners can also preserve their physiological characteristics better than males, as demonstrated by the low decoupling. Females demonstrate a greater proportional area of type I fibres, greater capillary-to-fibre ratio, greater volumes and densities of mitochondria, superior rates of oxidative enzyme activity [46, 47], have greater reliance on fat metabolism than males [48], and may thus be better protected from glycogen depletion. As a result, females may preserve muscular contractile function through better maintenance of glycogen [49], and propensity for greater proportion of fatigue resistance of type I fibres [46, 47]. Combined, whilst males will typically demonstrate a higher CS and better overall marathon performance, these factors may help explain why females were able to complete the marathon at a greater percentage of CS than males and did so whilst experiencing less decoupling.

### Limitations and Future Research Directions

For this study, we relied on a large dataset of recreational runners. Using such a large dataset allowed the exploration of decoupling characteristics during the marathon, and offers an insight as to whether the internal-to-external workload experienced during prolonged exercise contributes to explain marathon performance. However, when utilising this approach to use raw training data to calculate CS, it was not possible to verify if participants have performed a maximal effort, for example, checking whether $\dot{V}{\text{O}}_{2\max }$ has been attained during constant work rate trials [19]. Nonetheless, it is worth noting that this approach has previously been used to estimate CS with a low standard error of estimate (~ 8%) and to successfully predict marathon performance [15]. Data was used for ~ 4 months prior to a marathon event, and so it is likely that some activities included in the data set corresponded to maximal efforts through shorter races (e.g., 5 km) or higher intensity training sessions. Moreover, it has been demonstrated that extraction of data from training results in a high level of agreement with laboratory-based testing when estimating critical power, with low prediction errors (< 5%) [50]. Future research may wish to identify means of verifying maximal efforts to improve CS estimates from training data. It is also worth noting that the CS is an estimation of the upper boundary of the heavy intensity domain, and it was not possible to verify whether this represented the MMSS in the current study. It has been suggested that the CS may overestimate the MMSS relative to other methods and is highly dependent on the protocol used [11, 12]. However, the CS has been shown to closely represent the MMSS [14], and is widely regarded as an accurate tool to estimate of the heavy-severe domain transition [8, 13]. Furthermore, other methods used to approximate the heavy-severe boundary, for example, ventilatory thresholds, maximal lactate steady state, etc., were not permissible using the current approach.

To quantify internal workload, we used HR data, and it should be acknowledged that HR is likely to exhibit somewhat different kinetics to that of $\dot{V}{\text{O}}_{2}$ during prolonged exercise [43, 51]. Moreover, prolonged exercise can result in fluid loss due to excessive sweating and inadequate fluid replacement, particularly in hot environments. This imposes an additional cardiac strain, which results in a cardiovascular drift (i.e., increased HR, with concomitant reductions in $\dot{V}{\text{O}}_{2\max }$ [52]). Environmental conditions were not taken into consideration for the current analysis, but it is plausible that the decoupling of internal-to-external workload is increased in hot environments. Moreover, males and females may not be equally affected by exercise-induced dehydration [53]. A question that remains unanswered and warrants further investigation is whether durability traits are sensitive to training adaptations. We would also encourage further research to investigate whether training characteristics, such as training volume, intensity, or the distribution of training load, can influence durability. Nonetheless, the findings from the current study would suggest that training may be able to reduce the decoupling of the internal-to-external workload ratio.

## Conclusions

The internal-to-external ratio during a marathon was ~ 1.16, which represents a 16% increase in internal-to-external ratio over the course of the marathon, and was first detected ~ 25 km into the marathon. However, there was a large inter-individual variation in both the absolute magnitude of the decoupling and its onset. Importantly, both decoupling magnitude and onset were associated with performance, and the inclusion of these durability traits increased the precision of models of marathon performance by ~ 20% compared to those relying exclusively on CS and *D*′. Females had, overall, a better durability profile, as they exhibited lower decoupling in internal-to-external ratio. The data presented herein, therefore, suggest that appreciation of inter-individual differences in athlete durability may help improve understanding of an individual athlete’s performance capabilities in marathon running.

## Declarations

### Availability of data

The data supporting the findings of the current study are provided by Strava^®^ under a limited research license. The data are thus not publicly available. Requests to access these data should be directed to Strava^®^.

### Code availability

The code used to analyse the data is available upon reasonable request to Prof. Barry Smyth (barry.smyth@ucd.ie).
